# Analysis of codon usage patterns in *Hirudinaria manillensis* reveals a preference for GC-ending codons caused by dominant selection constraints

**DOI:** 10.1186/s12864-018-4937-x

**Published:** 2018-07-17

**Authors:** De-Long Guan, Li-Bin Ma, Muhammad Salabat Khan, Xiu-Xiu Zhang, Sheng-Quan Xu, Juan-Ying Xie

**Affiliations:** 1 College of Life Sciences, Shaanxi Normal University, Xi’an, 710119 Shaanxi People’s Republic of China; 2 School of Computer Science, Shaanxi Normal University, Xi’an, 710119 Shaanxi People’s Republic of China

**Keywords:** Leech, *Hirudinaria manillensis*, Codon usage bias, Evolutionary force, Transcriptome analysis, Genetic formation, Coding sequences

## Abstract

**Background:**

*Hirudinaria manillensis* is an ephemeral, blood-sucking ectoparasite, possessing anticoagulant capacities with potential medical applications. Analysis of codon usage patterns would contribute to our understanding of the evolutionary mechanisms and genetic architecture of *H. manillensis*, which in turn would provide insight into the characteristics of other leeches. We analysed codon usage and related indices using 18,000 coding sequences (CDSs) retrieved from *H. manillensis* RNA-Seq data.

**Results:**

We identified four highly preferred codons in *H. manillensis* that have G/C-endings. Points generated in an effective number of codons (ENC) plot distributed below the standard curve and the slope of a neutrality plot was less than 1. Highly expressed CDSs had lower ENC content and higher GC content than weakly expressed CDSs. Principal component analysis conducted on relative synonymous codon usage (RSCU) values divided CDSs according to GC content and divided codons according to ending bases. Moreover, by determining codon usage, we found that the majority of blood-diet related genes have undergone less adaptive evolution in *H. manillensis*, except for those with homologous sequences in the host species.

**Conclusions:**

Codon usage in *H. manillensis* had an overall preference toward C-endings and indicated that codon usage patterns are mediated by differential expression, GC content, and biological function. Although mutation pressure effects were also notable, the majority of genetic evolution in *H. manillensis* was driven by natural selection.

**Electronic supplementary material:**

The online version of this article (10.1186/s12864-018-4937-x) contains supplementary material, which is available to authorized users.

## Background

Codons are the basic genetic code of mRNA in all living organisms. There are 64 types of codons that encode 20 different amino acids; thus, codon degeneracy exists. In nuclear genomes, almost all amino acids, except for methionine and tryptophan, are encoded by two to six synonymous codons [1–3]. For different genes or genomes, the choices of synonymous codons are non-random, which is known as codon bias. Codon biases are specific to a given organism and can be influenced by GC content, gene expression levels, and gene lengths [4, 5]. Additionally, codon usage patterns may affect the mRNA biosynthesis, translational elongation speed, protein folding, and other biological functions downstream of expression [4–8]. Given the substantial biological effects of different codon patterns, identifying these patterns in a given gene or genome is important for understanding the molecular mechanisms of expression and for revealing the effects of long-term evolution on a genome.

Currently, the most popular and widely accepted hypothesis that explains codon usage bias is mutation-selection balance, which proposes that codon usage reflects the combined effects of three evolutionary forces: mutation pressure, selection constraints, and genetic drift within a population [4, 6, 8]. In general, mutation pressure tends to shuffle A/U and G/C pairs to cause nucleotide composition bias, selection constraints lead to preferences for codons that maximise protein production efficiency in highly expressed genes, and genetic drift eliminates codon changes across generations as a result of immigration and emigration at the population level. The codon usage pattern of a given organism reveals the balance between these three forces and by employing widely used methods, such as effective number of codons (ENC) plots and neutrality plots, the driving forces and approximate contribution of each force to codon usage can be determined. Previous studies have shown that codon biases in microbes are generally driven by mutation pressure, as exemplified in *Xanthophyllomyces dendrorhous*, Zika virus, and *Escherichia coli* [9–11]. Among invertebrate animals, selection constraints are the major influence on codon usage, as demonstrated in taxa such as *Drosophila melanogaster*, *Bemisia tabaci*, and *Taenia pisiformis* [7, 12, 13]. These studies demonstrate that characterising codon usage and other relevant indices provides simple and intuitive strategies for examining complex evolutionary forces in many different species.

*Hirudinaria manillensis* (Hirudinidae: Arhynchobdellida: Annelida) is an ephemeral, blood-sucking leech of medium body size that parasitizes mammals [14]. An adult *H. manillensis* may consume more than 10 ml of blood per host and can cause substantial and ongoing pain. Even after the leech abandons the host, this pain can persist as bleeding continues due to the anticoagulants (e.g., hirudin) secreted by the salivary glands of the leech [14–16]. The remarkable anticoagulant characteristics of *H. manillensis* secreted proteins have attracted much interest and the animal is considered a useful model for designing natural coagulation inhibitors and anticoagulant drugs [15, 17, 18]. Progress in molecular biology has generated interest in the analysis of *H. manillensis* mRNA with the aim of revealing details about their nutritional requirements and mechanisms of expression of anticoagulants. It would be of great value to generate more reliable coding sequences for new gene discovery; furthermore, the genetic processes revealed by their codon usage patterns will further contribute to determining the effects of long-term evolution on the genetically diversified and unique physiological behaviour of medical leeches. Such information on codon usage could be applied to biological research as a molecular tool to edit or optimise mRNA sequences as a way to control gene expression.

Because of the interest in leech biology and codon usage patterns and the paucity of related research, we conducted transcriptome sequencing of *H. manillensis* with the objective of providing the first detailed genomic information of the species. All identified unigenes and their annotations have enriched the current genomic sequences and can serve to further identify potential anticoagulant genes. We also attempted to determine the general driving evolutionary forces and to describe in more detail the differential evolutionary status for the identified anticoagulant genes. All high confidence unigene coding sequences (CDSs) were included to analyse codon usage bias, and the related indices and functional classifications. The general pattern of codon usage reflects the evolutionary process, and the differences in evolution between different metabolic mechanisms can be assessed by comparing these patterns. The results reported here will improve our understanding of the genetic architecture and mechanisms in *H. manillensis,* and will link the unique codon usage patterns of *H. manillensis* to specific genetic or genomic functions to help build a genetic profile for future research on the species*.*

## Results

### Nucleotide composition differs between codon positions in *H. manillensis*

**Fig. 1:**
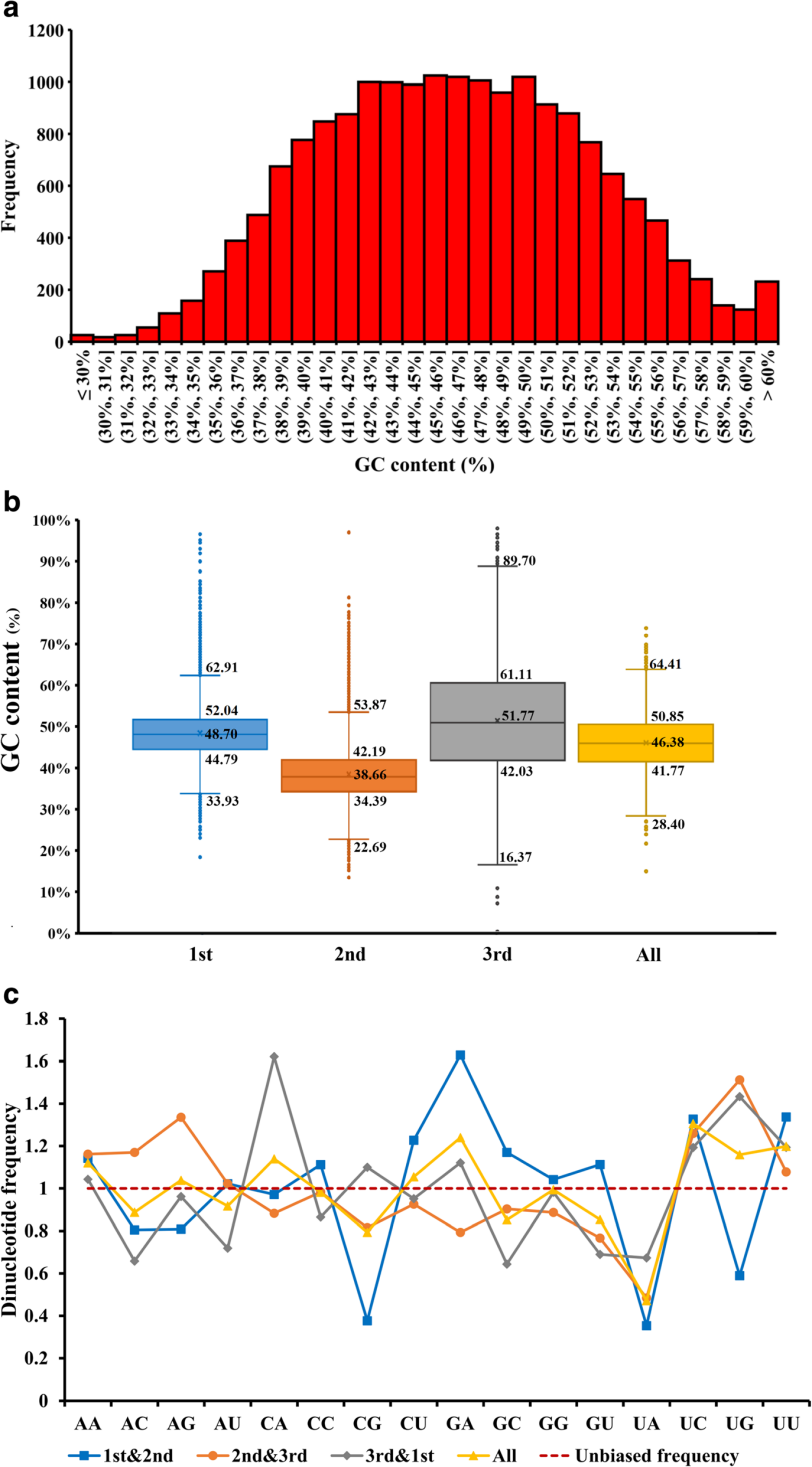
Base composition of *H. manillensis*. **a** Distribution of CDSs with different GC content levels in of *H. manillensis*; (**b**) Box plot indicates GC content variation in different codon positions (1st, 2nd and 3rd represent the first, second and third codon position, respectively) and overall genome (All). **c** Dinucleotide frequency plot in different combination of different codon positions (1st&2nd, 2nd&3rd and 3rd&1st) and overall genome (All), unbiased frequency means the theoretical frequency when all dinucleotides were presented equal, the curve located at 0.0625 (1/16)

The error-corrected transcriptomic data retrieved from *H. manillensis* samples measured 14.06 Gb and was comprised of more than 47 million raw reads. From these data, we assembled 115,132 raw transcripts and obtained 82,159 high-confidence transcripts. Using these 82,159 transcripts, we then identified 29,132 unigenes and 18,000 CDSs longer than 300 bp. The average lengths of the transcripts, unigenes, and CDSs were 1573.1, 1025.7, and 992.7 bp, respectively. Overall, the four nucleotides were asymmetrically represented in the CDSs. Adenine (A) was the most represented (29.3%), cytosine (C) was the least represented (23.0%), and there was little difference between guanine (G) (23.9%) and thymine/uracil (T/U) (23.8%) content. The GC content of the CDSs was 46.9%. To better understand the nucleotide base composition in *H. manillensis*, we measured the number of CDSs with different GC content levels. Almost all CDSs contained 30–60% GC content (Fig. 1a); thus, CDSs having GC contents lower than 30% or higher than 60% were considered as CDSs with extreme GC content. We further divided the 30–60% range into three smaller intervals and measured the number of CDSs attributable to each interval. The 42–50% interval contained the most CDSs, with numbers approaching or exceeding 1000, followed sequentially by the 50–60% and 30–42% intervals. This result corresponds with and to some extent explains the overall nucleotide composition of all *H. manillensis* CDSs measured*.*

We also measured the GC content at different codon positions (first, second, and third) in the CDSs. The composition at the first codon position was similar to that of the overall nucleotide composition. The average GC content and the range between upper and lower quartiles were the lowest in the second codon position, and the average GC content and the range between upper and lower quartiles and boundaries were the highest in the third codon position (Fig. 1b).

We performed a dinucleotide analysis on four CDS datasets at different codon positions (1st & 2nd, 2nd & 3rd, 3rd & 1st) to define the lower order patterns in the CDSs. All start and stop codons were removed because they are constrained by other rules. The ratios calculated using the observed frequencies over their theoretical frequencies, which are the mathematical products of the corresponding nucleotide content, are shown (Fig. 1c). For all four datasets, the dinucleotides were largely variable and unrelated to the expected unbiased frequency line. In the dataset that contained all CDSs, eight dinucleotides (AA, AG, CA, CU, GA, UC, UG, and UU) were more prevalent than the rest, with UA having the lowest frequency. Similar results were found in the 2nd & 3rd and 3rd & 1st databases, with only slight differences between them, while the ratios varied dramatically and lacked CG, UA, and UG pairs in the 1st & 2nd dataset. From the dinucleotide plot results, we observed that the various compositions of the sequences were not artefacts of different combinations of asymmetric nucleotide content. The sequences were formatted on the upper functional scale.

### Relative synonymous codon usage (RSCU) patterns in *H. manillensis* CDSs

Among the 61 synonymous codons identified (stop codons were excluded), 30 were more frequently represented with an RSCU > 1 (Table 1). The RSCU value of the codon GCC, which encodes Ala, was the highest. We divided these 30 codons by their ending bases and found that they ended with A (6), U (6), C (13), and G (5) in differing degrees. *H. manillensis* transcripts clearly preferred codons with GC-endings, particularly those ending with C, over the other bases. Furthermore, we wanted to locate the preferred/avoided codons and, therefore, emphasized those codons that had extremely high (> 1.25) or low (< 0.75) RSCUs. We found that codons such as GCC, CUC, and GUC were highly preferred and codons such as CUA, UUA, and AUA were particularly avoided in the CDSs that we evaluated. These highly preferred/avoided codons deviated from the neutral frequency value (equal to 1), which showed a preference for codons that ended with the GC dinucleotide.

**Table 1:** Relative synonymous codon usage (RSCU) values of coding sequences (CDSs) in *Hirudinaria manillensis*

AA	Codon	RSCU	AA	Codon	RSCU	AA	Codon	RSCU	AA	Codon	RSCU
Ala	GCU	1.06	His	CAC	1.111	Pro	CCA	1.16	Ser	UCG	0.907
	GCG	0.438		CAU	0.889		CCC	0.883		UCU	1.062
	**GCC**	**1.39**	Ile	AUU	1.074		CCU	1.134	Thr	ACC	1.134
	GCA	1.113		AUA	0.69		CCG	0.823		ACA	0.938
Cys	UGU	0.81		AUC	1.236	Gln	CAA	0.962		ACG	0.895
	UGC	1.19	Lys	AAA	0.981		CAG	1.038		ACU	1.032
Asp	GAU	0.966		AAG	1.019	Arg	AGA	1.025	Val	GUU	1.12
	GAC	1.034	Leu	CUA	0.365		AGG	0.975		GUG	1.029
Glu	GAG	0.837		**CUC**	**1.335**		**CGA**	**1.379**		**GUC**	**1.409**
	GAA	1.163		**CUG**	**1.334**		CGC	0.944		GUA	0.442
Phe	UUU	0.915		CUU	0.967		CGG	0.702	Trp	UGG	1
	UUC	1.085		UUA	0.546		CGU	0.975	Tyr	UAC	1.15
Gly	GGU	0.972		**UUG**	**1.454**	Ser	AGC	1.061		UAU	0.85
	GGG	0.536	Met	AUG	1		AGU	0.939			
	GGC	0.948	Asn	AAC	1.087		UCA	0.957			
	**GGA**	**1.544**		AAU	0.913		UCC	1.074			

Notes: All codons except for stop codons were included in this analysis. AA: amino acids. Codons with RSCU > 1.25 are shown in bold, codons with RSCU < 0.75 are shown with underlined text

We also analysed the ENC values in *H. manillensis* collected from the CDSs*.* The ENC values varied, ranging from 32.8–58.2, which indicated different trends in codon preferences between genes; however, using the convention of ENC = 35 to delineate strong and weak codon usage bias, we identified only three strongly biased CDSs (ENC values < 35). The majority of CDSs had a weak bias, confirmed by the average ENC value of 52.2. Such high ENC values suggest that almost all types of amino acids were used in protein synthesis, and that in most cases the preference toward particular codons had not developed in the genome and that codon bias is weak in *H. manillensis*.

### Codon preference is determined by multiple genetic factors within the *H. manillensis* genome

**Fig. 2:**
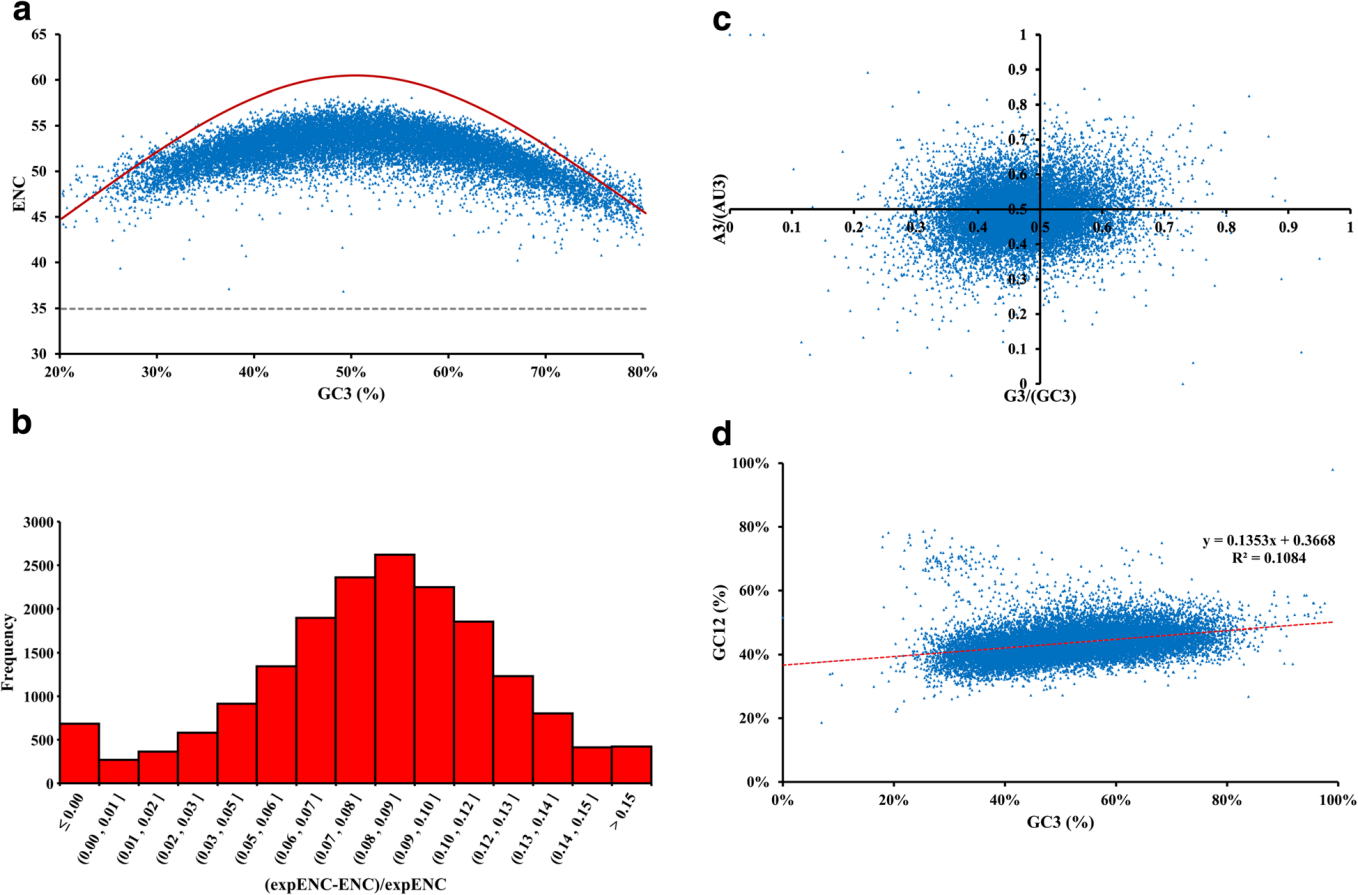
Characterise of evolutionary forces in *H. manillensis*. **a** ENC-GC3 plot. The solid line represents the expected curve when codon usage bias is only affected by mutation pressure. **b** Frequency distribution of ENC. **c** PR2-bias plot. Using the values of A3/(A3 + U3) against G3/(G3 + C3). **d** Neutrality plot analysis of GC12 and GC3 contents

As shown by the ENC plot, the vast majority of ENC values for *H. manillensis* CDSs were between 40 and 60, and these values were not significantly changed by different GC3 values. Almost all points in the plot were distributed away from the expected ENC curve in a pattern unrelated to the curve (Fig. 2a). The ENC value of 35 is highlighted in the plot, and the CDSs that have an ENC less than 35 are located under the highlighted line, they all have high GC3 values. Thus, we determined that codon usage bias was ubiquitous in *H. manillensis* and compositional constraint was not the determining factor driving codon preference, indicating that the pattern of codon usage in *H. manillensis* was caused by multiple factors as well as the composition of the ending dinucleotides. These other independent factors could be related to natural selection such as expression level or protein length, which may be more important than those related to mutation pressure.

To provide a more accurate assessment of the deviation between observed and expected ENC values, the formula (ENCexp − ENCobs)/ENCexp was used to calculate the ratios and to describe the degree of variation. Ratios between 0.06 and 0.12 were the most frequent in our dataset, while ratios between 0.08 and 0.09 were relatively uncommon, as determined by calculations using 2500 permutations (Fig. 2b). This result showed that, in most cases, the observed ENC values were slightly lower than the expected ENC values and differed by less than 10%. Therefore, other factors have influenced codon usage bias formation in *H. manillensis*, which has caused a reduction in usage complexity of approximately 10%.

### C/U to a/G balance in third codon positions is disrupted in *H. manillensis* coding sequences

Although it has been reported in analyses of mutation pressure that GC and AU occur in pairs at third codon positions, our results indicate that the proportions of G3 and C3 (or A3 and U3) are in fact unequal in *H. manillensis*. Evaluating the direction of such bias within AU and GC pairs can provide more detail about the forces that drive deviation from neutral mutations. We used a Parity Rule 2 (PR2) plot to measure these biases in *H. manillensis* CDSs (Fig. 2c). Most of the points in our plot fell between 0.3 and 0.7 on both axes, indicating an overall low bias toward either A3/U3 or G3/C3 in *H. manillensis*. Furthermore, we divided the points into four quadrants centred on 0.5 to compare the quadrants and found that the third quadrant (in which the ratio of A3/AU3 and G3/GC3 < 0.5) contained substantially more points than the other quadrants, whereas the first quadrant contained the fewest points. These results indicated that CDSs in *H. manillensis* had a slight yet noticeable preference for U and C at the third codon position. Therefore, the balance between AU and GC has been disrupted in *H. manillensis*.

### Natural selection is the dominant evolutionary force driving *H. manillensis* codon usage bias

To further clarify the driving forces behind, and the precise proportions of mutation or natural selection pressures contributing to, codon usage bias in *H. manillensis*, a neutrality plot was constructed using GC3 and GC12 values with a mean GC content at the first and second codon positions (Fig. 2d). Many points in the graph were distributed away from the regression curve and the R^2^ of the equation was low. These results indicated that the overall correlation between GC12 and GC3 was not strict, and that GC12 and GC3 had different degrees of modification. Additionally, the slope of the estimated equation revealed that mutation pressure accounted for a minority of the effects observed (13%), whereas selection constraints accounted for the majority (approximately 86%).

### ENC values and GC content are unrelated to differential gene expression

**Fig. 3:**
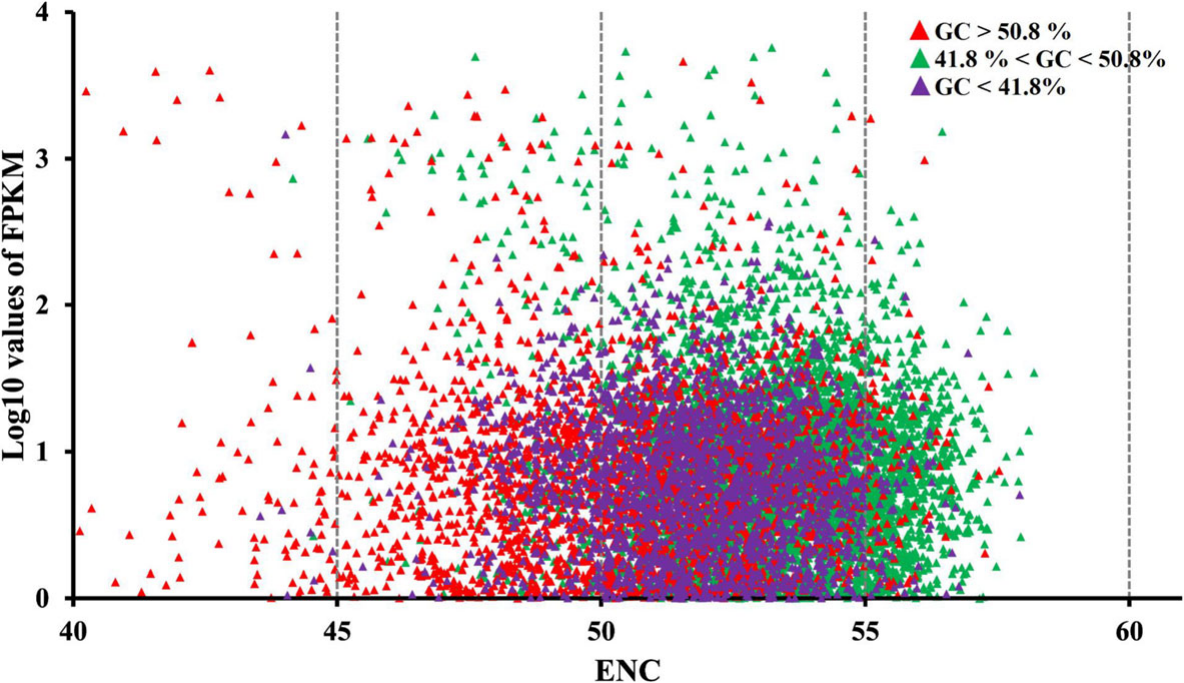
GC content and codon usage bias variation among expressed unigenes. All CDSs with observed FPKM values above were taken and transformed using log10, respectively. Dots with different colors represent their different GC content, while red represent GC > 50.8%, green represent 41.8% < GC < 50.8%, purple represent GC > 50.8%

One of the advantages of analysing transcriptome data is that the expression level of all genes can easily be obtained; therefore, earlier methods that used codon adaption index (CAI) values to predict expression levels among genes are no longer necessary. To explore the relationship between codon usage and gene expression levels, we selected CDSs of different genes that had fragments per kilobase million (FPKM) values above 1, and we plotted their log10 values against their ENC values. The purpose of calculating log 10 values in this situation is to condense the broad range of FPKM values. To further investigate the effects of genetic composition bias, we also labelled different points that represented the GC content of these CDSs. The upper and lower quartiles of the overall GC content in *H. manillensis* were 50.8 and 41.8% (Fig. 1b; above), respectively; therefore, we set three intervals for categorising different CDSs: GC < 41.8%; 41.8% < GC < 50.8%; and GC > 50.8% (Fig. 3). The three CDS groups with mostly overlapping distributions were found near the abscissa axis. The non-overlapped regions indicated that most genes with ENC < 50 were from the GC < 41.8% group, and the genes with ENC > 55 were from the 41.8% < GC < 50.8% group. Effects of selection were observed, indicating that the nucleotide substitution favoured GC and led to a stronger codon usage bias. However, on the vertical axis, neither the ENC values nor the GC content were separated from the FPKM values. This result indicates that the gene expression levels were generally unrelated to codon modifications, and that selection constraints of expression efficiency were not present. These results seemingly contradict each other, and to overcome this conflict we inferred that the overall expression profile was responsible. We reasoned that, to maintain general biological homeostasis, there must be many genes that are highly expressed but are not codon-modified due to their vital functions. Under selection, these genes would maintain their functions and eliminate codon changes that would jeopardise these functions. Therefore, genes with codon modifications would have higher expression efficiency due to adaptive selection forces, but the effects would be hidden among other genes.

### Principal component analysis (PCA) reveals that RSCU patterns are related to GC and codon-end binucleotide content

**Fig. 4:**
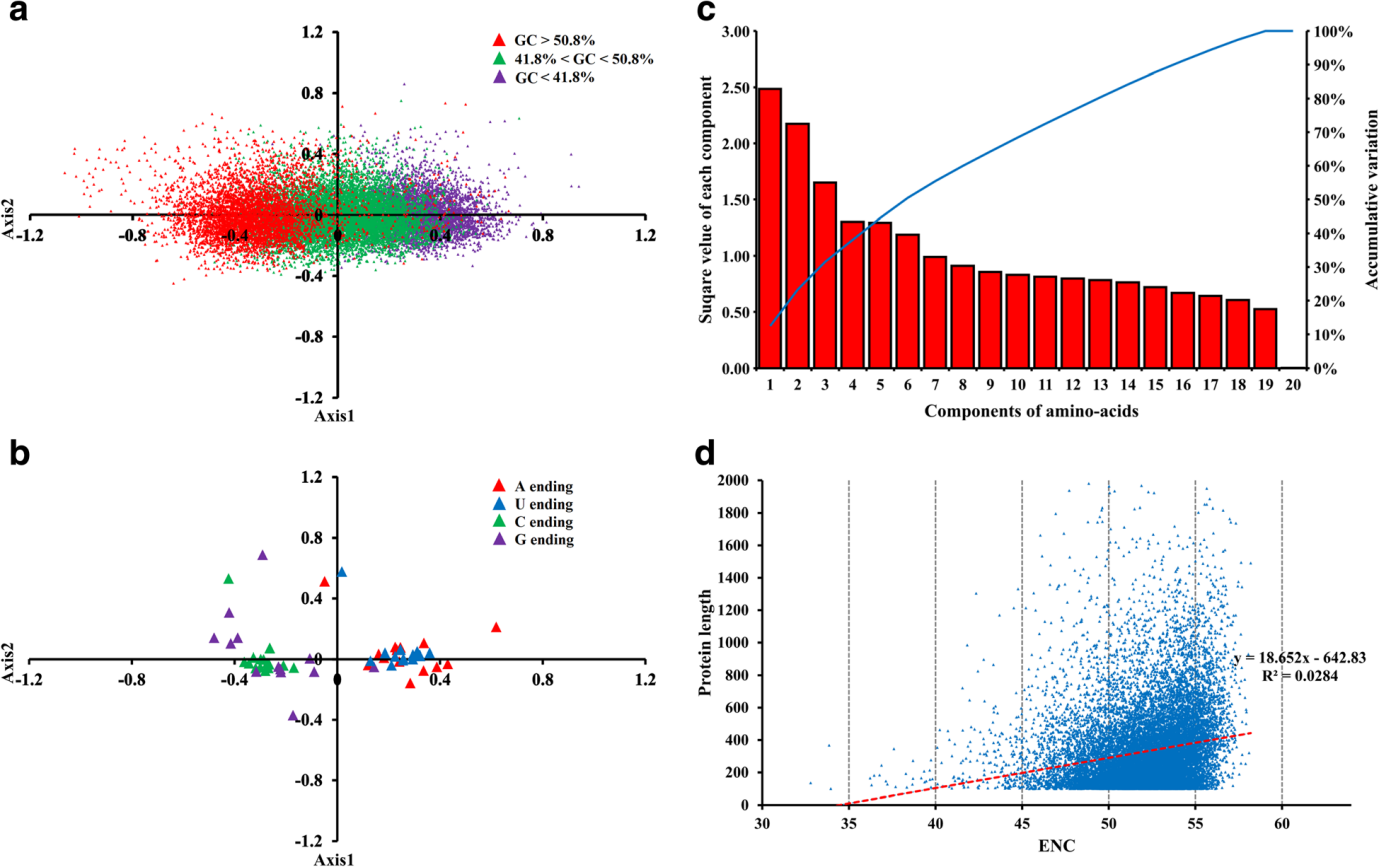
Effects of different indices on codon usage bias. **a** Principal component analysis of RSCU values of unigenes, the color of purple, green and red show different GC content. **b** Principal component analysis on RSCU values of codons. Different ending bases were shown in red, blue, green and purple. **c** The relative first 20 factors from principle component analysis based on the amino acid proportions. **d** Plot of ENC value variation against different protein length

In our PCA of the RSCU values, the first two axes accounted for 19.91 and 4.83% of the total variation. The primary axis (axis 1) accounted for much more variation than axis 2, indicating that the overall codon usage pattern described by RSCU was straightforward. To investigate the effects of genetic composition, the labelling intervals of GC < 41.8, 41.8% < GC < 50.8%, and GC > 50.8% were also used to divide the CDSs into three groups (Fig. 4a). The three CDS groups were distributed along axis 1, indicating that the primary axis was the only characteristic that could account for the differences between the groups. Considering the positions of the groups, and following the direction from negative to positive along axis 1, the three groups were distributed sequentially and discretely (i.e., GC < 41.8%, then 41.8% < GC < 50.8%, followed by GC > 50.8%). Taken together, these results indicate that the different GC content usages generated distinctly different distributions of RSCU values, and that effects of nucleotide composition were clearly present in the codons.

To investigate RSCU usage patterns among codons, codon positions were also analysed using PCA. All 59 codon triplets, excluding stop codons and codons encoding Met and Trp, were divided into four groups according to the ending base (Fig. 4b). Distribution regions were overlapped between codons ending in A and U, or codons ending in G and C. The AU and GC pairs had similar RSCU usage patterns, indicating general effects of mutation pressure on codon usage. Further comparisons within each group indicated that pairs ending in AU were more concentrated than pairs ending in GC. Additionally, G-ending codons were extensively distributed across three quadrants, which is the most varied among all codons. RSCU usage values for C- and G-ending codons occurred independently of codon type, indicating that the C- or G-favouring usage bias was driven predominantly by selection constraints. RSCU usage patterns are formed according to complex evolutionary forces; considering that the RSCU analysis showed that C-ending codons were generally preferred by evolutionary pressures (Table 1), these results suggest that the direction of these forces may inherently favour G and C bases.

### Codon usage bias is affected by protein length

Usage trends in amino acid composition were analysed using PCA with 20 axes (Fig. 4c). The accumulative variance in the first six axes explained over half of the total amino acid variation and, accordingly, they were considered to be the principle components. The first axis accounted for 12.41% of the total variation and the second axis accounted for 9.27%. The amino acid primary axis was not correlated with either the ENC value (*r* = 0.017; *p* > 0.05) or the RSCU primary axis (*r* = 0.19; *p* > 0.05); thus, amino acid composition does not affect codon usage patterns in the *H. manillensis* transcriptome.

Weak linear correlations were found between protein length and ENC (*r* = 0.03, *p* > 0.05), and between protein length and RSCU primary axis (*r* = 0.09, *p* > 0.05). Unlike amino acid composition, we observed two patterns when we analysed the variation between different proteins: short proteins had varying ENC values, and there was more variation in protein length with higher ENC values. Codon usage bias is affected by protein length, and we have inferred that they might be negatively related. To test our hypotheses, we plotted ENC against protein length, yielding results that supported our hypothesis (Fig. 4d). There was a Gaussian distribution between protein length and ENC. Short protein products that contained less than 200 amino acids had the greatest variation in ENC values. The majority of data points were distributed between ENC values of 50 and 55. Of the factors used in this analysis, length explained the greatest variation in ENC values, with results ranging by a factor of 10 between the lowest and highest values. These observations indicated that protein length affected codon usage as a result of selection constraints. Although an overall weak codon bias was not exclusive to proteins of certain lengths, longer protein products had a weaker codon usage bias. Conversely, shorter proteins did not particularly affect the codon usage; therefore, their usage patterns were predominantly shaped by other selective forces.

Hydrophobicity and aromaticity of the amino acids for each protein were not correlated with ENC (*r* = 0.005 and 0.028, respectively; *p* > 0.05) or with the primary RSCU axis (*r* = 0.078 and 0.133, respectively; *p* > 0.05). This result agreed with our finding that amino acid composition had little impact on codon usage. Thus, it is unlikely that codon usage bias in *H. manillensis* is influenced by hydrophobicity or aromaticity. Furthermore, because hydrophobicity and aromaticity determine the way proteins fold and how structures are configured in living organisms, we inferred that selection pressure during the protein folding process has little genomic effect. Thus, protein length affects codon usage, albeit only through its effects on translation.

### Comparison of codon usage bias between different anticoagulant genes reveals their evolutionary status

**Fig. 5:**
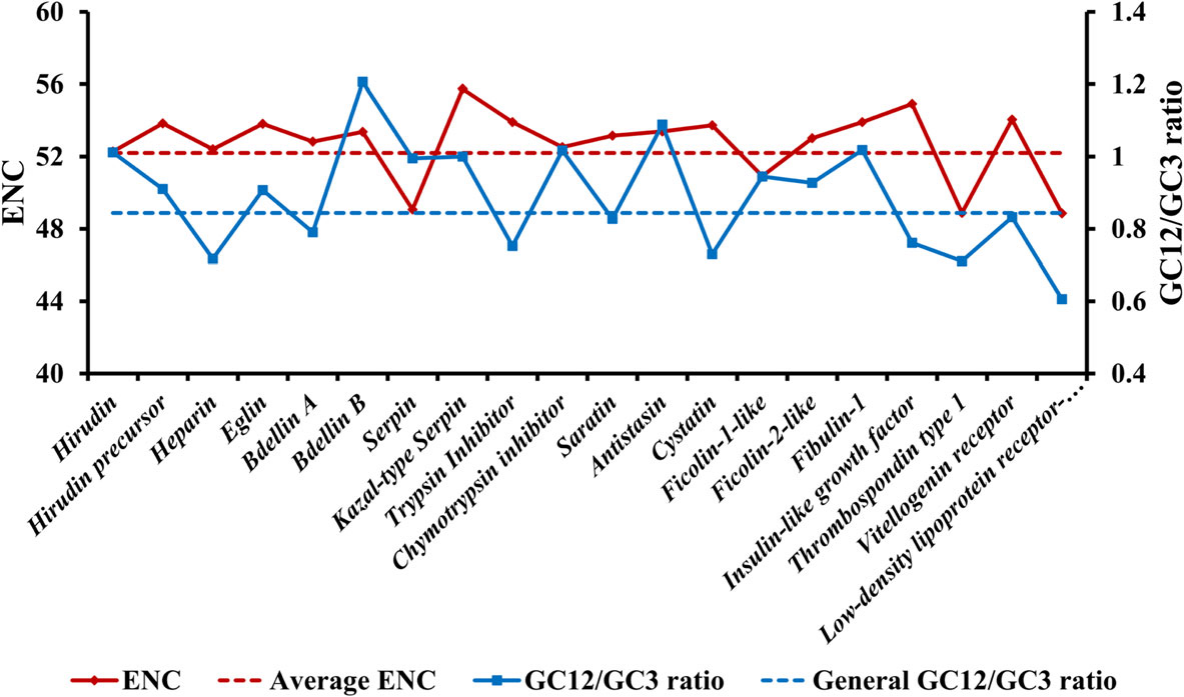
Comparisons of GC12/GC3 ratio and ENC values between different functional categories. The average ENC and general GC12/GC3 values were used to illustrate the differences of these CDSs from the overall tendency, the average ENC value is obtained as 52.2 and general GC12/GC3 ratio is calculated to be 0.83

In our study, unigenes were annotated by at least one database, which was helpful for analysing their various functions. *H. manillensis* has a blood-rich diet and its anticoagulant factors are a scientifically and medically interesting feature; therefore, we selected 20 CDSs from genes reported to encode anticoagulant products. Their codon usage patterns, which were calculated as GC12/GC3 ratios and ENC values, were compared to the average values obtained from all CDSs to measure the effects of different evolutionary states of anticoagulant-related mechanisms. In particular, we aimed to examine whether the anticoagulant genes had more adaptive modifications, which would help in understanding the evolutionary mechanism that drives anticoagulant characteristics. The results showed that 20 unigenes that encode genes such as hirudin, heparin, and eglin have plotted data points that are above the average GC12/GC3 ratios and ENC values, indicating generally weaker codon usage than average (Fig. 5). The effects of modifications on the codons for most direct anticoagulant genes were relatively relaxed, indicating that they were less adaptively evolved than were other parts of the genome, and that the genomic profile for the blood diet in *H. manillensis* matured before this species evolved from its ancestor. Moreover, we found that the four genes that encode serpin, filcolin-like 1, thrombospondin type 1, and low-density lipoprotein receptor-related protein had lower ENC values than average. In particular, thrombospondin type 1 and low-density lipoprotein receptor-related protein also had a GC12/GC3 ratio below average. Interestingly, for these genes, a homologous sequence can be found in the genome of the host species, indicating an evolutionary force that has driven these genes to emerge and correspond to their host-specific adaption.

## Discussion

### Transcriptome sequencing enriches our understanding of the *H. manillensis* genome and identifies codon usage patterns

Next generation transcriptome sequencing technology can greatly increase the quantity of known mRNA sequences for a given organism [19, 20]. To date, fewer than 100 mRNA sequences from the *H. manillensis* genome have been reported to NCBI. Thus, our work, which retrieved 115,132 transcripts, greatly expands the molecular database for *H. manillensis.* After strictly filtering these transcripts, the identified CDSs provide an overview of exons and the proteins that they produce, effectively identifying molecules with direct biological functions.

Our results demonstrate that AT(U)/GC nucleotide usage differs between the three codon positions, and that these composition differences likely drive the overall codon usage bias in *H. manillensis*. However, codon bias was limited overall. Because many codons are used during translation, we further hypothesised that extreme codon usage bias may only develop in particular or limited conditions. Additionally, our results show that G and particularly C are more common in third codon positions than expected. Third-position codon bases are directionally substituted, possibly because of effects from natural selection or mutation pressure. Identifying which of these forces drive the preference for C-ending codons helps elucidate the roles different forces can play in the evolution of *H. manillensis*. Because synonymous codons are not equivalent by their nature, the preference for ending bases may be caused by directional substitution, as a result of selection constraints acting against a background of mutation pressure. Our results indicate that overall expression levels regulate genomic composition and modify codon usage bias. The direction of this bias manifests as increased expression that leads to stronger codon usage bias and more mutational substitution toward GC. Thus, selection plays a dominant role in shaping the codon usage bias in *H. manillensis*. Future efforts to identify factors that lead to codon usage bias via base selection, including expression levels, protein lengths, and amino acid proportions, will provide greater understanding of the formation of particular codon usage patterns.

Transcriptome data have additional advantages in the analysis of codon usage [2]; for example, the expression levels of genes can be detected and described in terms of FPKM values. According to the mutation-selection-drift theory, selection constraints can direct a preference toward codons that promote translation efficiency. Values such as FPKM can be essential for these types of analyses; thus, previous studies that were unable to obtain absolute results were obliged to use one of several theoretical indices to measure expression rates, such as CAI, codon bias index (CBI), or frequency of optimal codons (Fop) [20–23]. Although these indices are statistically valid and have helped describe the formation of codon usage patterns in several species [4, 12, 24, 25], their use remains problematic because of their dependence on the correct identification of optimal codons, which vary between genes and organisms and do not always promote translation efficiency. For instance, in humans, cell- and tissue-specific tRNA composition is determined solely by translational elongation rates and optimal codons have no effect on this determination [26–28], which limits the ability to make certain predictions from transcriptional data. Accordingly, we conclude that using FPKM values (actually, the log10-transformed values) for genes obtained from transcriptome data generate more accurate results and that these values also help refine existing theories for the calculation of codon usage indices.

Additionally, transcriptome data allow for detailed identification of unigenes and CDSs at various magnitudes, which in turn assists in identifying genomic effects on biological functions. We identified unigenes by comparing homology with Nr, GO, KEGG, and several other databases, and removing those that lacked annotations to ensure that each CDS was described in at least one unigene database [29–32]. These CDSs could be divided into different categories and comparison of these categories could be used to measure the differential effects of the relevant selection pressures in *H. manillensis*. We plan to use these comparisons in future studies to identify and characterise economically valuable components of the *H. manillensis* genome, such as genes that contribute to anticoagulant production. The results of these future analyses could lead to viral-mediated gene therapy to harness their potential to benefit human health.

### Unique *H. manillensis* codon usage patterns

Characterising codon usage patterns in *H. manillensis* was one of the principle aims of our study and the data provide the basis for future analyses that will detect evolutionary pressures. The overall codon usage bias that we detected in *H. manillensis* was not extreme, but did show a preference for GC-ending codons, particularly C-ending codons. Similar biases have been detected in other species, but mostly invertebrates [12, 13, 19, 22, 33–35]. In the leafhopper *Homalodisca coagulata,* 11 out of 15 preferred codons ended in C [12], and in the cestode *T. pisiformis*, 17 out of 32 preferred codons were GC-ending, with 12 ending in C [13]. Similar patterns across species that suggest long-term evolution in codon usage could be regulated by common biological mechanisms in invertebrates. The factors affecting codon bias in other species are likely to contribute to the codon bias that we characterized in *H. manillensis* as well. The uniqueness of codon usage in *H. manillensis* could be related to the different evolutionary forces that contributed to the preferences for specific usage effectors, including nucleotide composition, gene expression, and protein length.

### Formation of codon usage patterns in *H. manillensis*

Almost all previous studies on metazoans that used ENC and neutrality plots support the conclusion that selection has a greater effect on codon usage than mutation pressure [6–8, 12, 13, 19, 24–27, 34–36]. In these cases, ENC values deviated from expectations. GC12 was positively correlated with GC3, and GC12 was lower than GC3, which are consistent with the results we presented for codon usage patterns in *H. manillensis*. The ENC values for the CDSs we examined did not follow the standard expENC curve, but GC12 did correlate with the shallow but linear slope (0.3201) of GC3. Although some ENC values fell on the expected curve, we believe that they do not refute the overall pattern of random codon usage in this species; the random codon usage that we identified was most likely caused by strong selection over long periods of evolution, which is the driving force behind existing codon usage bias in *H. manillensis*.

In addition to identifying selection as the driving force behind codon usage bias in *H. manillensis*, we measured the effects of the factors that produce selection pressure on codon usage patterns. The overall effect of gene selection is the maintenance of efficient gene expression; however, selection pressure from previous translation processes, also known as the translation elongation rate, or certain subsequent protein products could also contribute to this selection process. Previous studies have shown that both conditions occur in different species. In many viruses such as bovine coronavirus or Zika [10, 37], protein products are not contributing factors, whereas in plants like *Zea mays* [38] the effects of proteins products are significant. In our study, we investigated factors such as nucleotide content, expression levels, amino acid composition, protein length, hydrophobicity, and aromaticity to evaluate their relationships with codon selection. Of these factors, only protein length had selective effects on codon usage; however, the effects of protein length were strictly determined by CDS length and overall, attributable to the translation elongation rate. Thus, we conclude that codon usage bias in *H. manillensis* is shaped only by translational mechanisms, the final protein products of which may be sufficiently conserved to be excluded from evolutionary processes.

### Tendency toward GC-ending codons in *H. manillensis*

In *H. manillensis*, we identified preferred codons that used all four bases; however, the distribution of ending bases was uneven and favoured GC-ending codons. One possible explanation for this result is that preferred codons undergo selection that modifies ending bases from AU to GC over long-term evolution. According to the mutation-selection-drift theory, preferred codons are selected because they better match the tRNA genes that promote translation efficiency [4]. Because we identified selection as the dominant force in *H. manillensis* evolution, we hypothesised that GC-ending codons would increase in frequency with increased gene expression. This speculation was largely supported by our comparative analysis of ENC and GC content between the highest and lowest expressed genes in *H. manillensis*. We suggest that richer and more stable GC content in codons facilitates a more efficient use of tRNA in *H. manillensis*, as tRNA is the foundation of genetic mechanisms. As in many invertebrates [13, 19, 34, 35], preferred codons tend to include GC, and we suggest that this tendency is exhibited by other species and could be strengthened further by evolution. Understanding these preferences facilitates the editing and promotion of the in vivo expression of target genes in *H. manillensis*.

### Codon usage patterns in different anticoagulant genes expressed in *H. manillensis*

Many previous studies have examined the molecular mechanisms and identified several functional genes in different types of leeches [16, 18, 39–41]. The genes that encode hirudin, heparin, bdelin, and eglin have been characterised and are well known as typical anticoagulant-related sequences in medical leeches [39–41]. The results of our gene annotations were largely similar to those reported in previous studies, which confirmed these as functional genes. Using these genes as examples enabled us to evaluate their evolutionary status in *H. manillensis.* Codon usage is predominantly affected by environmental forces and can be used to infer the differential effects of long-term evolution. The overall codon usage pattern could be representative of the current genetic nature of this species, while the draft status of these genes is more or less modified by differentiation during adaptation to specific environmental conditions. Thus, we inferred the existing adaptions in these functional anticoagulant genes and found that relaxed selection likely occurred. The majority of blood-diet-related genes have undergone less adaptive evolution in *H. manillensis*, except for those with homologous sequences in the host species. Based on these findings, we further suggest that the basic evolutionary mechanisms of genes in *H. manillensis*, especially genes common in other phylogenetically related leeches, have promoted genes that have undergone less adaptive selection than their genome due to their mature molecular mechanism. Only genes that have newly participated in evolution will be modified by stronger selection, as revealed in their codon usage.

## Conclusion

This study characterised the transcriptome of *H. manillensis*, a leech known for its medicinally significant anticoagulant properties. The nature and functional pathways of all genes were identified in this species, and the effects of different evolutionary factors on codon usage were characterised and measured. As with other invertebrates, we found that in *H. manillensis* there is a preference for GC-ending codons, particularly those ending in C, and concluded that the primary contributing force to this preference is selection rather than mutation. These conclusions contribute to our knowledge of the *H. manillensis* genome, improve our understanding of invertebrate genomics, and provide further validation of genomic tools for indirectly measuring evolutionary processes.

## Methods

### Sample acquisition

The *H. manillensis* samples used in this study were obtained from ponds in Hechi City, Guangxi Province, China. *H. manillensis* is an invertebrate and is not endangered; there are no restrictions on the capture of *H. manillensis* individuals*.* Following taxonomic identification, samples were stored in liquid nitrogen until RNA extraction.

### *H. manillensis* transcriptome sequencing

To obtain the transcriptome, an Illumina HiSeq 2500 high throughput sequencing platform was used for sequencing (Illumina, San Diego, CA, USA). Two cDNA libraries, designed T05 and T06, were derived from the extracted total RNA of two *H. manillensis* individuals and were used for sequencing. Libraries were prepared with a NEBNext UltraTM DNA Library Prep Kit (New England Biolabs, Ipswich, MA, USA) following the manufacturer’s instructions. A total of 14.06 Gb of sequence data was collected for T05 and T06, comprising 36,998,868 clean reads with Q20 and Q30 values higher than 99.0%. Reads were deposited in the NCBI database (Project number: PRJNA382869, Accession numbers: SRX2735687 and SRX2735688).

Sequence assembly was conducted in Trinity 2.4.0 (http://trinityrnaseq.github.io/) using default settings [20]. Transcripts were identified using Transdecoder, which is a function within Trinity [42]. To avoid instrumental errors, identified transcripts were confirmed using BLAST (https://blast.ncbi.nlm.nih.gov/Blast.cgi) and only those that were present in both T05 and T06 were retained [43]. BLAST results of the common sequences were saved into one file and 115,132 transcripts were obtained. Further, to exclude all possible contaminants such as bacterial or other types of RNA (e.g., tRNAs and rRNAs) that could bias the results, we built a high confidence dataset from the transcripts by blasting our results to the transcriptome data of *Poecilobdella javanica* (SRR5429898), a species that is phylogenetically close to *H. manillensis*. With the blastx E-value set to 1^e-50^, we obtained 82,159 transcripts with an average length of 1573.1 bp. The unigenes were identified using these highly confident transcripts and the final number of unigenes that we identified was 29,132.

### Identification and annotation of CDSs

Prior to identifying and annotating the CDSs, all obtained unigenes were first annotated using BLAST in the following databases: NR (NCBI non-redundant protein sequences) [29], Pfam (Protein family) [44], COG/eggNOG (clusters of orthologous groups of proteins) [30, 45], KEGG (Kyoto Encyclopedia of Genes and Genomes) [31], and GO (Gene Ontology) [32]. The GO database can be used to identify the biological functions of genes in different functional categories, and the KEGG pathway database can be used to identify the series of interactions caused by genes that produce new molecules or lead to other changes within cells. The BLAST parameters x, p, and r were set under E-value = 1^e-5^. Unigenes that were not annotated were excluded from further analysis. The remaining unigenes were used in the identification of CDSs using ORFfinder (http://www.geneinfinity.org/sms/sms_orffinder.html). To ensure the quality of these coding sequences, low quality sequences that were smaller than 300 bp, contained internal N gaps, or included too many internal stop codons were removed. Finally, a total of 18,000 qualified CDSs were identified and annotated in at least one database.

### Codon usage bias and related index analysis

CodonW 1.4.4 (https://sourceforge.net/projects/codonw/) [46] and DAMBE 5.2.30 (http://dambe.bio.uottawa.ca/DAMBE/dambe.aspx) [47] were used with the default parameters to calculate the essential raw values used in this study. For RSCU values, a standard codon table was chosen. The ENC values, which represent the capacity for codons to encode amino acids and are negatively related to codon bias, were calculated automatically in CodonW while calculating RSCU. These two parameters were used to describe codon usage patterns. Several indices such as the overall GC content, GC content at the first, second, and third codon positions (GC1, GC2, GC3), amino acid composition, protein length, aromaticity (AROMO) values, and general average of hydrophobicity (GRAVY) values were also calculated. In particular, reliable nucleotide content for single nucleotides and dinucleotides, amino acid composition, and protein lengths were conducted in DAMBE and validated using CodonW. GRAVY and AROMO values represent the mean frequency of hydrophobic and aromatic amino acids among gene products, respectively, and were calculated in CodonW. All these results can be accessed in Additional files 1, 2, and 3. Additional file 1 contains the values of ENC, expected ENC, GC content, protein length, GRAVY, and AROMO. Additional file 2 contains the RSCU values of each sequence and the first 2 PCA factors. Additional file 3 contains the proportion of amino acids in each sequence and the first 2 PCA factors.

### ENC plot

ENC plots are scatter diagrams that compare observed ENC against expected ENC (expENC) for different GC3 (G or C in the third codon position) values. When observed ENC values fall on the curve of expected ENC values for a given sequence, mutation pressure is the sole force acting on third-position bases in that sequence. To make this plot, we submitted 1000 strictly increasing GC values from 0.001 to 1.000 to DAMBE to obtain their expENC values. These values were then used to draw a standard curve; a smooth curve was selected to connect these value points. We then plotted all of the points that represented the observed ENC and GC values from each sequence to the curve to make the final plot.

### Neutrality plot

The obtained GC3 and GC12 (mean GC1 and GC2) values were plotted on the lateral and vertical axes, respectively, to produce a scatter diagram for the neutrality plot. The formula between the GC3 and GC12 was estimated with a linear model using SPSS 22.0. Neutrality plots are primarily used to assess the mutation pressure bias that affects codon usage bias. When the slope is 1, codon usage bias is driven solely by neutrality. The effects of other forces such as selection constraints can generally be inferred when the slope deviates from 1.

### Parity rule 2-bias plot

The parity rule 2-bias plot was constructed using the values obtained from the formulae A3/(A3 + U3) and G3/(G3 + C3). These data were visualised in a scatter diagram with a plot root of 0, and thus all data points were distributed into four quadrants.

### Principal component analysis (PCA)

RSCU values are particularly useful for describing codon usage bias because they indicate the precise frequency of the appearance of each synonymous codon in a given coding sequence. However, using a matrix of RSCU values directly can cause difficulties in the identification of the major driving force of codon usage bias, and the connections between factors involved. PCA can be used as a dimensionality reduction tool to significantly reduce the data complexity by using principal components to reflect overall patterns. With this method, a matrix comprising complex RSCU values can be transformed into just a few major axes. Furthermore, by combining and illustrating other codon usage-related factors, the effects of these target factors are shown more intuitively at the macro level.

PCAs were defined using RSCU values and the proportion of amino acids as factors in SPSS 22.0 (SPSS Inc. Software, Chicago, Illinois, USA). We included all 61 codons (excluding three stop codons) and 20 amino acids in the CDSs to compare GC content and codon usage with the RSCU values. We selected four principal components among the RSCU values and two major groups were correlated with GC content to assess whether GC was a contributing factor to RSCU values. The PCA of RSCU values in the CDSs of unigenes was used to separate codons, whereas the PCA of the proportion of amino acids was used to measure cumulative inertia and identify patterns in amino acid usage. PCA initially produces a series of orthogonal axes that can classify trends that represent variation in the data, with each subsequent axis contributing a smaller proportion to the variation. PCA locates each codon or amino acid on these axes and the location is the combination of columns (codons or amino acids) and rows (genes) and is highly distinguishable between different groups.

### Statistical analysis

All mathematical analyses, including PCA, correlations, and *t*-tests were calculated in SPSS 22.0 (SPSS Inc. Software, Chicago, Illinois, USA). Figures were generated in Excel 2016 (Microsoft, Redmond, WA, USA) and SPSS 22.0.

## Additional files

Additional file 1: The values of ENC, expected ENC, GC content, protein length, GRAVY, and AROMO. This file contains the calculated values of ENC, expected ENC, GC content, protein length, GRAVY, and AROMO. Different columns represent different value types, and their specific meanings were shown at the beginning of each column. (XLSX 1818 kb)

Additional file 2: The RSCU values of each sequence and the first 2 PCA factors. This file contains all the calculated RSCU values of each sequence and the first 2 PCA factors. The stop codons were not included and thusly this file has 62 columns. The beginning of each column shows the specific meanings of the different value types. (XLSX 6743 kb)

Additional file 3: The proportion of amino acids in each sequence and the first 2 PCA factors. This file contains all the obtained proportion of amino acids in each sequence and the first 2 PCA factors. All 20 types of amino-acids were shown in this file, and thusly it has 23 columns. (XLSX 4402 kb)

## Abbreviations

AROMO: Aromaticity
CDS: Coding sequences
COG/eggnog: Clusters of orthologous groups of proteins
ENC: Effective number of codons
FPKM: Fragments per kilobase million
GC1: GC content at the first codon positions
GC2: GC content at the second codon positions
GC3: GC content at the third codon positions
GO: Gene Ontology
GRAVY: General average of hydrophobicity
KEGG: Kyoto Encyclopedia of Genes and Genomes
Nr: NCBI non-redundant protein sequences
PCA: Principal component analysis
Pfam: Protein family
RSCU: Relative synonymous codon usage
